# Investigate knowledge management technology implementation for supporting decision making in Ethiopian health sectors

**DOI:** 10.1186/s12911-021-01507-9

**Published:** 2021-05-05

**Authors:** Mniyichel Belay, Amare Desta, Steve Smithson, Million Meshesha

**Affiliations:** 1 Department of Information Science, Jimma University, Jimma, Ethiopia; 2 Information and Knowledge Management, Queen Mary University of London (QMUL), London, UK; 3 Department of Management, London School of Economics, London, UK; 4 School of Information Science, Addis Ababa University, Addis Ababa, Ethiopia

**Keywords:** Healthcare, KM technology, Clinical decision making, Ethiopian hospitals

## Abstract

**Introduction:**

Knowledge management technology is a key tool for facilitating and improving the quality of health care delivery in hospitals. However, the use and implementation of this technology is not an easy task. This study aims to investigate a knowledge management technology implementation and use in Ethiopian hospitals, with a particular reference to Jimma University Specialized Hospital.

**Methodology:**

Information about challenges of knowledge management technology implementation was gathered using qualitative research methods, through conducting semi-structured interviews. Purposive sampling method was applied to select fifty-four participants from a well-defined population. Findings were first validated, according to published literature and research works, and then sorted into three main areas, such as current usage of KM technology to enhance and improve decision-making, factors affecting the implementation of KM technology

**Results:**

This study revealed that the overall level of KM technology implementation in Jimma University Specialized hospital is still low.

**Conclusion:**

Implementation and use of KM technology to improve the quality of hospital health services is needed. Thus, it suggested that hospital managers should make much more effort to develop a strategy and policy on the implementation and use of KM technology so that the hospital could improve the quality of healthcare services.

## Introduction

Nowadays, knowledge management technology is the major contributor to improve the service of healthcare. In organizations with an environment of unorganized information resources, technology has become the major issue for decision-makers. Technology can change the health sector and it also becomes an essential issue for the sustainable development of every organization [1]. For example, doctors and medical practitioners are overwhelmed by newly generated knowledge and it is difficult for them to separate this knowledge into groups and store valuable information, discovering the appropriate information where they need it. In order to be able to identify the solution quickly, they must use and implement knowledge management technology. Newly created knowledge will strengthen the decision-making process and help not only to build the solution on their expertise, but also to use the entire healthcare delivery services [2].

Knowledge management technology is referred to as the use of electronic media to manage health and medical care information of the patients [3]. It is an umbrella term to describe the full management of health information across the computerized systems and its secure exchange between consumers, service providers, government and quality entities, and insurers. Similarly, knowledge management technology has the potential to improve the quality, safety, and efficiency of healthcare [4].

According to [5], knowledge management technologies are the most abundant and widely used medical products in practice, providing many advantages to doctors and medical workers; but their implementation is difficult, particularly for sub-Saharan African countries. Even human and financial capital is limited to the use and implementation of knowledge management technologies [6], endorsed this concept and noted that developing countries are still struggling to uses and implement knowledge management technology due to lack of infrastructure, expense, and time and value analysis, lack of qualified labour, national policies, and health-related staff motivation. There is a shortage of appropriate technical skills linked to technology. Projects in the field of health technology lack the requisite budget for implementation. The period of time needed for the proper implementation of particular health technology is very weak.

Similarly [7], indicate that constraints to the implementation of technology in the hospital include a low budget for Information Communication Technology, poor infrastructure in the maintenance of health services, unreliable electricity supply and insufficient human resource capacity. Emdadulhaque et al. [7] stated further the major barrier to technology implementation is the failure of healthcare information systems (HISs) to interoperate in order to distribute information concerning different standards among the institutions in the healthcare sector.

Today in Ethiopian health sectors, the main barriers in implementing knowledge management technology with the clinical process are related to strategic context, organizational approaches, environmental and technological barriers. Asemahagn [8] reported that KM technology implementation in Ethiopian hospitals are still inadequately conceptualized in such a way that tools do not meet the complex needs of patients, professionals and organizations. For example, the benefits of knowledge management technology are dependent on transferring knowledge across healthcare organizations. However, implementing KM technology with healthcare and the accessibility of knowledge are the main challenges observed in Ethiopian health sectors. Ethiopia has thus made progress in creating the conditions necessary to support and improve healthcare with the help of knowledge management technology; though significant challenges remain [9].

Furthermore, in Ethiopia health sector setting, the basic challenge remains the awareness of the importance and the potentials use of KM technology in health care. Besides, inadequate training for employees on KM technologies and up-to-date medical research, work-flow changes, lack of IT infrastructure, the unwillingness of clinicians to use KM technology on daily activities, lack of senior management support. Poor quality of managing knowledge and lack of fund to implement KM Technology, lack of communication between researchers and policymakers are some the challenges for implementing KM technology.

Therefore, the gap that called the researcher conducted this study explores challenges the implementation of KM technology with clinical processes for supporting decision making in the Ethiopian health sector. Thus, this paper aims to examine factors affecting the effective and efficient implementation of knowledge management technology that is useful to enhance and improve medical decision making. Accordingly, this paper describes the major constraints and barriers faced in implementing knowledge management technology effectively in the health sector of Ethiopia. It draws out good practices for using technology in the health sector and highlights priority needs and issues of relevance to policymakers. The paper also looks at emerging trends in the usage of technologies that are likely to improve healthcare services and identifies gaps in supporting decision making.

## Methods and materials

The study used a qualitative approach to investigate implementation of knowledge management technology for supporting decision making in Ethiopia health sectors. The reason behind using qualitative methods was to seek in-depth and condensed information about the study’s phenomenon [10]. Detail information about perceived challenges in the implementation of knowledge management technology in healthcare enables us to suggest better ways for improving the quality of decision making. Qualitative methods were used, through conducting semi-structured interviews, to collect opinions, experiences, and suggestions of stakeholders on this specific study area. Findings of the conducted interviews were first validated, according to published literature and research work, and then sorted into different challenges and opportunities.

For this specific study, the ethnographic methodology was used to investigate the challenges for knowledge management technology implementations in the clinical process of the selected hospitals in Ethiopia. The main reason for selecting the ethnographic methodology is that it enabled the researcher involved in collecting observational data of an intact cultural group of decision-makers. Ethnography has been described as the study of individual cultures and producing a “descriptive work from such research” [11]. Ethnography was selected for this study for two main reasons: firstly, it enabled the analysis of actual experiences when gathering empirical data and, secondly, it was most appropriate for studying different aspects of the research and comparing themes that emerged from the collected data from different sources and methods [12].

### Setting

The researcher identified two sites for conducting the assessment. The first one is a large size public hospital (Jimma University referral specialized hospital) and the second one is the Ethiopian Ministry of Health with a total number of fifty-four (54) from the two sites. Jimma University referral specialized hospital is located in Jimma town 352 km to Southwest of Addis Ababa; It was the only teaching and Referral Hospital in the Southwestern part of the country until recently. It runs an annual governmental budget of Birr 171.3 million with a bed capacity of 800, with 640 active. It has a total of 1837 staff currently including contract staff.

Some of the experts of knowledge management were selected from Ethiopian Minister of Health. IT professionals and knowledge managers participated in the study. These make them very familiar and in continuous contact with the hospital’s practical environment related to technology integration with the clinical process. The researcher observed that the respondents were appropriate to give their opinions and perspectives regarding issues raised in the study, which are related to challenges of implementing KM technology within hospitals services. Those participants for this study were to increase the validity and objectivity of the findings and to make use of different opinions in order to reach a better understanding of the situation.

### Study participants’ sampling

Purposive sampling was used to identify the Study participants’ in the study site. This allowed us to focus on a limited number of participants that have direct access to the study area and working environment. The participants were selected depending on the objectives of the study and the questions that we are trying to find answers. Anwar-ur-Rehman Pasha and Pasha [13] noted that the choice of a specific sampling technique depends on the objectives of the study and the questions that we are trying to answer. Table 1 below presents the study participants from the selected sites.

**Table 1:** Summary of sample participants in the study

Study site	Number of participants	Profession
Jimma University Referral Specialized Hospital	2	Managers/administrators
	6	Interns
	4	Surgeons
	4	ICT professionals
	8	General medical practitioners
	8	Pharmacists
	9	Nurses
	3	Radiographers
	4	Laboratory staff
Ethiopia Ministry of Health (MOH)	6	KM experts and HIT professionals
Total	54	

As shown in the above table the two managers were purposively selected from Jimma University specialized hospital: one clinical manager and one senior manager. Anwar-ur-Rehman Pasha and Pasha [13] said that different respondents had been selected to define the purpose and objective of the study. The two hospital managers were asked to take part in the study. The two managers involved in decision-making processes and are very concerned about the development of KM technology to enhance and improve decision-making. Similarly, the remaining participants were purposively selected Jimma University specialized hospital.

In the same way, six participants were purposively selected from the MOH. The practical activities of individuals are very well known and in continuous contact with the practical environment of the selected hospital and with the issues that healthcare workers face daily, which make them suitable candidates to give their opinions and perspectives on the subject of the study, which are factors affecting the use and implementation of KM technology in the hospital. Anwar-ur-Rehman Pasha and Pasha [13] noted that the choice of two sites and two groups of participants for this study was to increase the validity and objectivity of the findings and to make use of different opinions in order to gain a better understanding about the situation.

### Data collections and Data analysis

Semi-structured interviews were exploited for conducting this study. Although it is a time-consuming method, it allows the interviewees (i.e. the healthcare and ICT staffs) to express their opinions in a free and spontaneous manner [12,13]. The same open-ended questions were used with all the participants.

In this study, the researchers used a recorder and field note to collect audio, video and necessary data. Besides, collected audio or video data (e.g. recordings of interviews are transcribed into written form in field notes for further interpreting of data. Once all of the respondents are interviewed have been transcribed and checked, it is time to begin coding can be done by the researcher with hand on a softcopy and hard copy of the transcript. Mainly 3 with 31 sub item Codes or categories are classified or label for allocating research variables to the text compiled during a study.

Similarly, the researcher used a tool for qualitative analysis like NVivo. Zamawe [15] Supported that NVivo now forms an essential part of qualitative data analysis. Among others, NVivo saves researchers from ‘time-consuming’ transcription and improve the accuracy and speed of the analysis process. Also, excel with a check sheet and control chart was used to analyze, classify, sort and arrange data automatically.

Each interview started with a very general question about the participant’s name, age, current position and previous experiences to break the ice. Then followed three basic areas, such as the current practice of KM technology to enhance decision-making, tools selected for supporting decision making, and barriers for implementing KM technology with decision making.

The interviewees were signed a consent form. The interviews were recorded with permission from the participants and the interviewer afterwards conducted the interview. After conducting the interview, the preliminary data analysis was made. During data analysis paragraphs and sentences were coded, labeled and classified into (i) the use of knowledge management technology, (ii) types of technology used, and (iii) barriers to implementation of knowledge management technology for the clinical decision-making processes.

## Result and discussion

The results and the themes derived from the fifty-four interviews completed in this qualitative analysis are discussed in this section. Two of the managers who participated in this study were males and aged between 35 and 40 years. The majority of respondents, including KM experts and HIT experts from the Ministry of Health of Ethiopia (MOH), have relevant work experience in selected organizations. The study process aimed to describe the key problems and challenges facing healthcare workers with regard to the use and implementation of KM technology in selected hospitals in Ethiopia. It would help to define the best strategies that should be taken to improve the utilization and implementation of the technologies by highlighting these critical concerns.

Similarly, the interviewed experts highlighted different current activities and developments that contributed to improving the use of knowledge management technology for supporting decision making processes in a selected Ethiopian hospital. This paper first presents some positive highlights and next shows problems, challenges from the perspective of the interviewees. These include barriers identified and matters related to organizational structure, culture, managerial and professional skills, budget-related to it as well as technological infrastructure. Finally, the experts raised the central issue of coordination, collaboration, and governance and leadership, which are needed to improve the quality of healthcare service.

### Current trends in KM technology use for clinical decision making

In this section, the researcher wants to address respondents opinion on the currents trends of knowledge management technology used in Jimma University specialized hospital and Ethiopia ministry of health in terms of having organized knowledge to enhance and improve decision-making. Understanding of decision-makers, such as top-level manager, medical doctors is the main drivers for properly used technologies in this hospital. But it is well known that Ethiopian is the developing country which is highly needed to implement the technology to support knowledge management processes for improving decision making.

Respondents provide their opinion on the current trends of knowledge management technology used in Jimma University Specialized Hospital and Ethiopia Ministry of Health in terms of having organized knowledge to enhance and improve decision-making. Understanding of decision-makers, such as top-level manager and medical doctors is the main drivers for properly using technologies in this hospital. But it is well known that Ethiopia is one of the developing countries with much healthcare needs to implement the technology to support knowledge management processes for improving decision making.

The awareness of the importance of knowledge management technology for supporting decision making in Ethiopian hospitals, especially Jimma University specialized referral hospitals is one requirement to improve the quality of healthcare. Nurses replied that most of the decision-makers in hospitals are not familiar with the importance of medical technology. However, He strongly agreed that knowledge management technology could be a valuable tool for decision making and it should become a requirement for decision-making processes in the hospitals.

The notion of the importance of *knowledge management technology* was further emphasized by managers as noted below.

I think medical knowledge management technology is a good basis to support decision making as well as to improve the quality of health care. In Jimma University specialized referral hospital there is a responsible section to properly manage the created knowledge. However, this office is not well organized with knowledge management technology [R01].

Medical doctors and nurses were asked for their opinion on the current practice of supporting decision-making process with knowledge management tools in the hospital. Most of the decision-makers believe that it would be more effective if the decision-making process is more formal and structured to ensure that decisions are facts and evidence-based.

The use of technology *for supporting decision-making processes* is still very low in health sector. We always say that it’s very important for supporting decision making with suitable technology, but we still do not have awareness of how to use it [R32&R35].

Table 2 below presents common ideas from all respondents by classifying into the predefined three thematic areas.

**Table 2:** Thematic areas of analysis

No	Thematic areas	Common concepts
1	KM technology to enhance and improve decision-making	- Understanding - Current practice - Importance/usage - Tools
2	Types of technology used for supporting decision making in Ethiopian health sector	- Existing tools - Skills of using among the staffs - Professionals staff in the hospitals
3	The barrier to implementation of technology with decision making	- Organizational factor (human resources, culture and structure) - Environmental factors - Technological factors (accessibility, availability and)

### Types of technology used in the Ethiopian health sector to support decision making

This section addressed the kinds of technology used to facilitate decision-making in the Ethiopian health sector. It focused primarily on the current technologies or tools used to support decision making in this particular hospital.

There are currently numerous tools and technology used in the health sector to facilitate decision-making. These include databases; libraries built locally, different forms of artificial intelligence systems, including expert systems, system support for clinical decision-making, neural networks, fuzzy logic, genetic algorithms and agents of intelligence or software.

In addition, the types of technology an organization has been able to use effectively to enable and support clinical processes for evidence-based decision making. Nowadays, the Jimma University Specialized Hospital in Ethiopia has needed the implementation of unique technologies to help and enhance healthcare decision-making. In this link, the hospital information communication technology section head reported that

Currently, one of the key technologies that are used in hospitals is a human resource management information system. These tools enable to routinely generate human resources information for fact-based decision making [R11].

Furthermore, one of the staff of hospital information communication technology noted about lack of technology for managing knowledge.

No knowledge management technology can allow hospitals to build knowledge portals that can handle a substantial amount of information [R14].

Medical doctors were also asked about the type of technology that was chosen and used in hospitals to handle expertise and support decision-making. Consequently, their answer is summarized as follows.

Currently, there is no medical knowledge management technology that serves as a repository to have all the decisions made previously; so that they can use it as a reference in similar situations. However, hospitals tired to implement technology like an open clinical system for integrating the services of different department and sections [R09].

Similarly, the respondents from Information Communication Technology commented about the use of technology for enabling knowledge transferring in the clinical process*.*

There is no specified technology that can support the flow of knowledge with the clinical process but they only use the following computer-mediated communication such as electronic mail or conferences to create communication among staff members. [R12, R13 &R15].

Furthermore, most of respondents revealed that the individual staff skill on the use of technology is limited. Even we still don't use everything the technology offer, especially the technology related to managing knowledge for supporting decision making.

Ethiopian Ministry of Health has a plan to implement a healthcare information system in hospitals. Electronic medical record (EMR) systems and personal healthcare records (PHR) have been developed and deployed, transforming the customary patient paper-based record system. Even patient’s diagnostic data and treatment information have been converted into an electronic format that can be accessed by medical staff within a hospital and other partners to assess a variety of test results and provide assess a variety of test results and provide treatments.

### Barriers to implementing KM technology with clinical process

This section addressed several challenges identified as use and implement of knowledge management tools and technologies in the clinical processes. The challenges comprise the fears that available of captured knowledge and the problem with a store, reuse, disseminate or transfer for the decision making. The most important barriers are categorized related to organizational factors (human resources, culture/structure and finance), environmental and technological constraints.

The health sector faces many challenges in properly implementing KM technology with the clinical process. Accordingly, the interviewees identified many critical barriers for effective implementation of KM technology in health sectors.

The most organizational barriers are categorized into human resources, organizational culture, organizational structure, and overemphasizing technology. The most critical barriers identified from the interview were summarized in Table 3 below.

**Table 3:** Critical barriers to effective implementation of knowledge management technology related to organization factors

Main barriers	Specific barriers
Human resources management	- Lack of skill in the full profession - Lack of awareness of KM technology provisions - Absence of employee training - Absence of employee motivation - Absence of employee empowerment - Employees resistance to transfer knowledge
Organization structure and culture	- Poor management support - Poor organizational structures - Lack of leadership - Poor organizational culture - Insufficient planning - Lack of well-formulated strategy and policy
Finance	- Limited financial support for professional development - The poor financial investment of the organization - Security concerns - Insufficient IT investment

Most of the participants revealed that staffs are not strongly concerned with the implementation of systems, which requires overcoming expectations that it would be a simple replacement of their existing paper processes, as well as a continuous refinement of new electronic processes. Even, managers lack a strong commitment to organizational changes through the technology that can accompany the implementation of knowledge management technology to use it to its full capabilities.

Furthermore, the respondents from radiology, nurse and general practitioner asked about the weakness that prevents effective knowledge management technology implementation for supporting decision making across the hospitals. Their response is summarized as follows.

Knowledge management culture is still weak due to the following reasons, such as weak knowledge management procedures, weak information and communication technology infrastructure, weak networking activities, lack of financial resources to support knowledge management activities, lack of standards and tools for knowledge management, weak knowledge translation activities and low utilization of knowledge for decision making and policymaking [R17, R35 &R44].

On the other hand, environmental dimension involves factors and issues that are situated in the control of the hospital or the healthcare staff such as a limited number of healthcare staff needs to cope and handle on daily basis activities without adding further duties to their busy schedule. Similarly, several environment factors were captured by the current study (i.e. culture, financial support, top-level management support.

This research identifies the technology-related obstacles to implementing KM technology in healthcare. Most participants replied to the lack of technological resources: such as lack of information technology, in addition to personal computers and single-user access to existing healthcare activities, inadequate infrastructure, poor design and planning, poor networking and lack of maintenance and training.

## Discussion

Overall, participants describe that the decision making processes is not fully supported with knowledge management technology. Some of the reason is staff members still do not have awareness on how to use it for supporting decision making. Even*,* most decision-makers in hospitals are not familiar with the importance of medical KM technology. Pinheiro et al. [16] highlighted that the use of KM technology has not yet reached its full potential, since it is initially used by health administration for the decision-making process. In general, they are confronted with the challenges of using KM technology due to inefficient professional skills.

Despite limited studies of organizational factors related to human resources, particularly skills and knowledge of the use of KM technology in the developing world, particularly in Africa, several studies has shown that the skills and awareness of the use of KM technology among healthcare professionals are very low. In support of the above concept, Adane et al. [17] a report on computer and Internet usage by nurses from Nigeria's teaching hospital found that only 43% of nurses could use the technology. Likewise, only 33% of health professionals use technology for different purposes in Ethiopia.

Furthermore, based on the finding with observational methods the barriers identified as organizational factors for use and implementing KM technology in Ethiopia health sector were lack of top-level management support, Poor organizational structures, Lack of leadership, Poor organizational culture, Insufficient planning and Lack of well-formulated strategy and policy. Yagos et al. [18] confirmed that despite the other results of this report, many obstacles appear to obstruct the implementation and use of technology in healthcare. This includes insufficient technical knowledge and skills of health workers; weak administrative support, poor organizational structures, lack of leadership, poor organizational structure and lack of technology policy.

Generally, most of the respondents also revealed that successful implementation of knowledge management technology in clinical processes are important to improve decision making and thereby enhance organizational performance within health sectors. However, the level of awareness of implementing and use of knowledge management technology in support of decision-making processes is still insufficient.

The study shows that Ethiopia is looking towards the implementation of KM technology to improve its healthcare service. Towards this, there exist several opportunities for supporting knowledge management implementation using technology, though there are some barriers related to human resources development, structure/culture of an organization, technological and financial barriers [19] listed the following barriers to the implementation of KM technologies in healthcare. These barriers are decomposed into the following categories, i.e. Technological, finance, national policy, organizational structure and culture barrier framework. They boldly state that the key obstacles to implementing new KM technologies are required professional and qualified human capital.

Similarly, Schreiweis et al. [20] also identified the challenges for integrating technology in healthcare such as (1) organizational, (i.e. structure, culture and human resources) (2) environmental and, and (3) technological barriers. From the finding, the lack of technological resources: such as inadequate infrastructure, poor design and planning, poor networking and lack of maintenance and training are the main the technological barriers for use and implement KM technology in healthcare.

According to Baridam and Govender [21], inadequate infrastructure is responsible for the inefficiency in the healthcare delivery in healthcare services. Out of 247 respondents, 59% (144) believed that it was certain that the inefficient healthcare delivery within the organization could be attributed in part to the absence of technological infrastructures.

Therefore, from the above comparison of results from the finding and literature, we can understand that organizational, environmental and technological factors are the main barriers for use and implementing KM technology in health sectors.

## Theoretical framework

Bordoloi [22] noted that, as measured by the impact on organizational benefits, the implementation of knowledge management technology in healthcare delivery depends on the levels of organizational infrastructure (structure, culture, human resources), environmental and technological capability. In the context of healthcare delivery, a related knowledge management model proposed by Bordoloi [22] notes that essential KM technology contributes to improved decision-making which in turn contributes to organizational success in terms of quality, satisfaction and productivity. The significant role of the different factors that serve as critical factors for implementation KM technology is further inherent in the model [22] (Lee and Choi 2003).

**Fig. 1:**
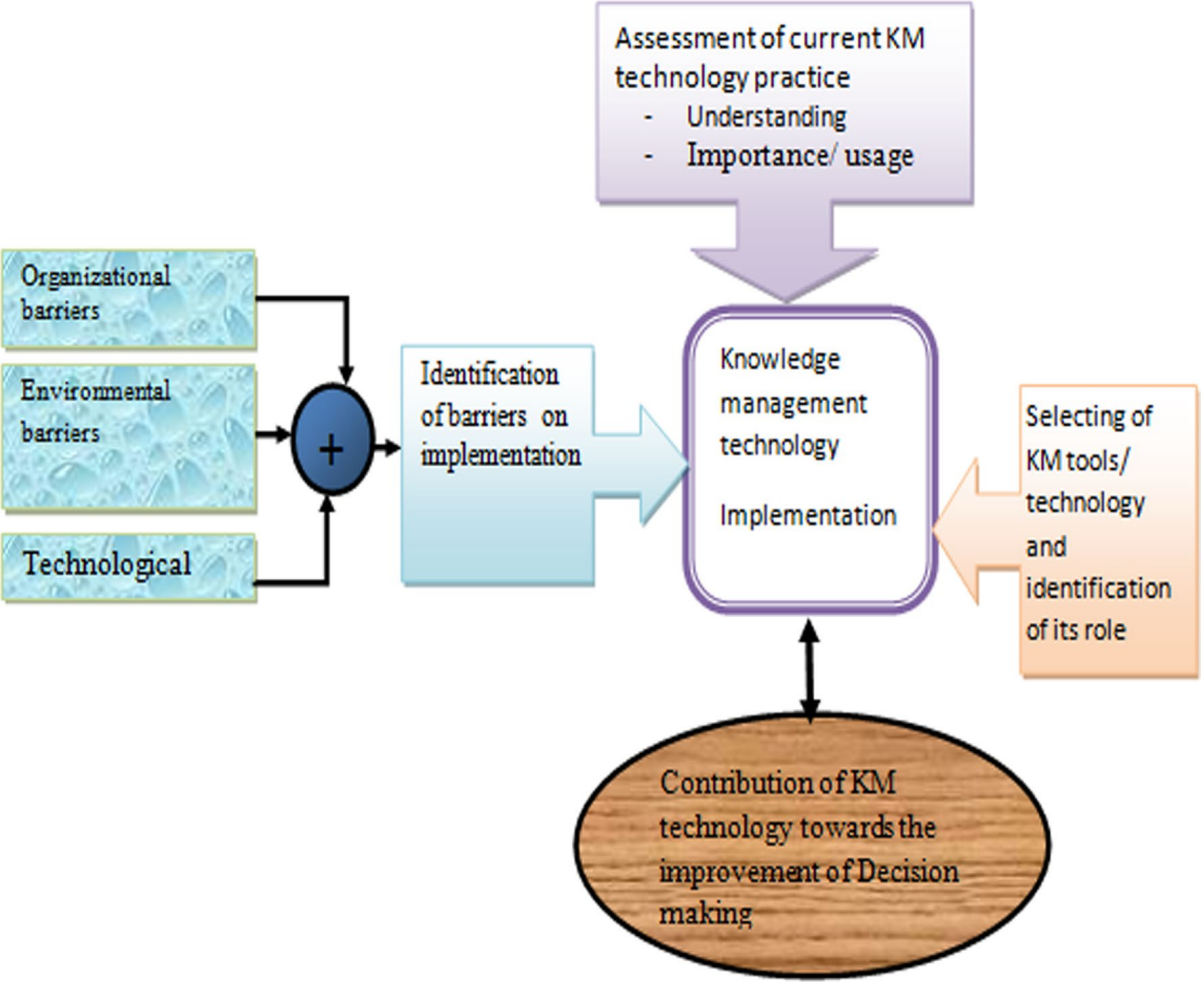
The theoretical framework for implementing KM technology for support decision making

Thus, based on the finding of the study and literature review of KM technology theories and models used to explore and study the implementation of technology in health care settings, the theoretical framework depicted in Fig. 1 is constructed.

Figure 1 presents the theoretical framework for implementing knowledge management technology that supports decision making in the health sector. On the way towards constructing the theoretical model, initial observation of the researcher generated different variables. The first one is variables that interact in the implementation of KM technology, such as Human resources management, Environmental with Organization structure and culture, Technological, Finance. The other is an assessment of current KM technology practice like Understanding, Importance/ usage. Finally, selecting locally and internationally developed KM tools, technology and identification of its role in the healthcare.

This study focuses on how use and implementation of knowledge management technology to facilitate decision making. A rounded corners box at the top labeled “assessment of current km technology practice” is shown the evolution of individual-level knowledge or understanding, beliefs, perception, and cognitive process that influence understanding, practice for the implementation of knowledge management technology. Similarly, a rounded corners box at the right, a labeled “selecting of km tools/ technology and identification of its role” represent the examined the possible and selected tools /technology confront with the knowledge management for improving the decision making in the clinical processes. besides, a box at left side labled “identification of challenge on implementation” shows challenges direct forwards to the characteristics of the organization such as organizational -level structure, culture and availability of resources, the technological character such as usability ad available of technical infrastructure that influences knowledge management implementation. Furthermore, an ellipse labeled “knowledge management technology implementation” represents the effects these changes have on the implementation of the technology.

In the current healthcare in Ethiopia, locally produced knowledge is not applied in improvements’ of service. Even a lot of knowledge is generated every day but not effectively used for evidence-based decision making. To this end, the implementation of KM technology has opened a new chapter for improving healthcare delivery. Besides, the above theoretical model, shown in Fig. 1 integrates the key factors discussed in the findings section that can lead to effective implementation of knowledge management technology to discover knowledge that already exists and organize it to for simplifying its accessibility so as to support decision making.

## Conclusion and recommendations

The main aim of this research is to identify the critical barriers for successful implementation of knowledge management technology from the viewpoint of healthcare professionals in Ethiopia health sectors. The findings from this study would make significant contributions both to theory and practice of sustainable information system implementations in Ethiopia relating to the health sector.

Identifying barriers in the Implementation of knowledge management technology are a hot issue in the health sector. Ethiopian health sector is still struggling for it and facing a lot of barriers like lack of infrastructure, cost, time and benefit analysis, lack of skilled workforce, national policies and motivation of health-related personnel. It is high time to recognize that evidence-based decision making is very helpful in enhancing the performance of health institutions. Enough professional skills related to technology usage are lacking. Knowledge management technology projects are lacking in the budget. The time period, which is required for a good implementation of the specific health technology, is quite long.

Finally, Ethiopian health sectors specifically Jimma University Specialized Hospitals should prepare the strategy and policy for supporting to implement knowledge management and simultaneously increase the awareness about supporting decision making with KM technology. Furthermore, the top-level management of the hospital should support knowledge management processes (i.e. knowledge transfer) with effective implementation of KM technology.

## Data Availability

The datasets used and analyzed during the current study are available from the corresponding author on reasonable request.
